# The role of motivation and emotions in physical education: understanding academic achievement and the intention to be physically active

**DOI:** 10.3389/fpsyg.2023.1253043

**Published:** 2023-09-20

**Authors:** Sebastián Fierro-Suero, Isabel Castillo, Bartolomé J. Almagro, Pedro Saénz-López

**Affiliations:** ^1^Faculty of Education, Psychology and Sports Sciences, University of Huelva, Huelva, Spain; ^2^Faculty of Psychology and Speech Therapy, University of Valencia, Valencia, Spain

**Keywords:** self-determination theory, control-value theory, physical activity, achievement emotions, academic performance

## Abstract

**Introduction:**

This study aims to understand how emotions and motivation influence the academic achievement of physical education (PE) students and their future intention to practice physical activity (PA). Despite the influence on student’s behaviors and the reciprocal associations between motivation and emotion, the number of studies addressing both constructs at the same level is very limited.

**Methods:**

A structural equation model was used with 799 students aged 11–17 years (*M* = 13.16; SD = 1.17).

**Results and discussion:**

The results showed that the teacher support of the basic psychological needs (BPN) predicted students’ BPN satisfaction, which in turn predicted their autonomous motivation and positive emotions, and negatively predicted their negative emotions. Finally, autonomous motivation predicted students’ intention to be physically active, whereas academic achievement was predicted by both autonomous motivation and emotions. We conclude that to better understand the consequences of PE classes, it is necessary to consider both constructs.

## 1. Introduction

Motivation and emotions are seen as prerequisites, mediators and even consequences of both learning and academic achievement, helping us to understand people’s behavior (Reeve, 2010; Pekrun et al., 2017). Both constructs, motivation and emotion, although they bear some similarity, are different (Løvoll et al., 2017). Motivation refers to the processes that give energy and direction to human behavior (Reeve, 2010), whereas, emotions are interrelated psychological processes consisting of cognitive, physiological, affective, expressive and motivational processes (Scherer, 2009). Emotions can influence people’s behaviors in various ways (Meyer and Turner, 2006). On the one hand, we find that the emotions experienced after an event help to maintain the pre-established motivation (Løvoll et al., 2017), indicating whether the situation is okay or it needs to change (Reeve, 2010). On the other hand, emotions can cause new directions in changing behavior (Løvoll et al., 2017), that is, they can function as a type of motive in themselves (Reeve, 2010). These internal processes (motivation and emotion) occur separately, because people differ in both emotional perception and in their motivational response to that perception (Roth et al., 2019). Although it does not occur in all cases, emotional behavior is usually associated with more impulsive behaviors while motivational behavior could be considered more deliberate (Roseman, 2013).

Theories that link emotion, cognition, and motivation have traditionally arranged the relationships between them hierarchically or chronologically (Meyer and Turner, 2006), that is, they have alternated between emphasizing motives versus emotions as sources of energy and control of behavior (Roseman, 2013). For instance, some authors (e.g., Izard, 1991; Lazarus, 1991) established the priority of emotions over cognitive or motivational aspects, a trend that has been studied over the years (Vandercammen et al., 2014). On the other hand, other authors have established the prioritization of motivation over emotion in the understanding of human behavior, relegating the emotional role to the consequences and not to the causes of behavior (Deci and Ryan, 1985; Ford, 1992). In the last decade, the interrelation between cognition, emotion and motivation has been demonstrated (Reeve, 2010). Specifically, in the educational field (Ramirez-Arellano et al., 2019; Järvenoja et al., 2020), this interrelation occurs within a complex system of co-regulation between students and teachers (Meyer and Turner, 2006). Thus, observation of teacher instructions associated with intrinsic or autonomous motivation (i.e., doing something because it is interesting or enjoyable) revealed the impossibility of differentiating between motivational and emotional aspects (e.g., smiling or joking on the part of the teacher when giving corrections) (Meyer and Turner, 2006), however, it is possible to differentiate in terms of individual perception (e.g., Ramirez-Arellano et al., 2019; Van Roekel et al., 2019). Therefore, the various emotional regulation processes exert an influence on volitional functioning, personal well-being and high-quality relationships (Roth et al., 2019), aspects that are closely related to those needs associated with motivational behaviors (Reeve, 2010). As a result, there is reciprocal associations between the two constructs (Van Roekel et al., 2019).

To better understand students’ behaviors (e.g., participation, academic achievement, disruptive problems, etc.), it is necessary to address both the emotional and motivational roles, given the interconnection between the two constructs (Roseman, 2013). Research on this topic has been requested by several authors (e.g., Meyer and Turner, 2006). Some studies have started to emerge in different educational contexts (e.g., Shao et al., 2020) however, most studies in the field of Physical Education (PE) that address student behaviors (e.g., Ulstad et al., 2016; Franco and Coterón, 2017; Cheon et al., 2018) have focused mainly on motivation, without taking into account the emotional component.

### 1.1. Self-determination theory

**Figure 1:**
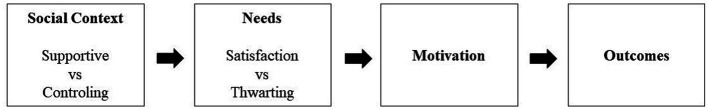
Illustrative model of self-determination theory.

As for motivation, Self-Determination Theory (SDT; Deci and Ryan, 1985; Ryan and Deci, 2017) has been postulated as one of the main theories to understand human behavior based on an illustrative sequential model (Vasconcellos et al., 2020; Figure 1). This theory suggests that people need to feel competent (to interact effectively with the environment), autonomous (to feel that they choose to perform the behavior) and related to others (to feel connected and respected by others), and that satisfaction of these three Basic Psychological Needs (BPN) is essential for the development of motivation, well-being and performance (Ryan and Deci, 2017).

The social environment plays an important role in the satisfaction or thwarting of these BPN. Specifically, in the school environment, in which young people necessarily spend a large part of their time, teachers (as authority figures), through their interpersonal style (autonomy supportive vs. controlling), satisfy or thwart those BPN (Haerens et al., 2015; Ryan and Deci, 2017). When the teacher uses a need supporting interpersonal style with the students, offering freedom in the choice of activities and encouraging students’ involvement in the decision-making process, the BPN will be satisfied, facilitating students to become more intrinsically and self-determinedly involved in their tasks (Haerens et al., 2015) and promoting performance and well-being. Whereas, if the teacher presents a controlling interpersonal style, behaving in a coercive, authoritarian way and exerting pressure on the students, these BPN will be thwarted, leading to lower motivation (Haerens et al., 2015), lower performance and greater discomfort, that is, more maladaptive consequences (Ryan and Deci, 2017).

SDT has been widely used in PE, showing to be effective in the application of intervention programs to increase students’ autonomous motivation (Kelso et al., 2020). It has also been shown to be useful for investigating different consequences associated with this motivation (Cheon et al., 2018, 2020), as well as the intention to be active in the future (Cheon et al., 2012; Franco and Coterón, 2017; Castillo et al., 2020), the levels of physical activity (PA) practiced in leisure time (Castillo et al., 2020), academic achievement in PE (Cheon et al., 2012; Ulstad et al., 2016) or some emotions like enjoyment (Franco and Coterón, 2017). Therefore, SDT has taken emotions as consequences of behaviors.

### 1.2. Control value theory of achievement emotions

**Figure 2:**
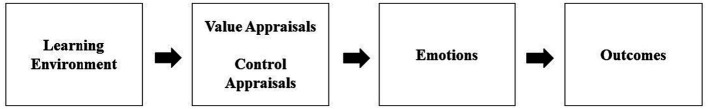
Illustrative model of control value theory of achievement emotions.

The evidence on emotions in PE is limited (Simonton and Garn, 2018) with most of the studies published on this topic being very recent. Among them, there is a clear tendency to use Control Value Theory of Achievement Emotions (CVTAE) (Pekrun, 2006) as the main theory to approach the study of emotions in the context of PE (e.g., Simonton and Garn, 2018, 2020; Fierro-Suero et al., 2020a, 2023; Zimmermann et al., 2021). CVTAE, which analyzes emotions from a cognitive-social perspective (Figure 2), maintains that achievement emotions are critical antecedents of control appraisals (competence beliefs, self-efficacy expectations, attributions of achievement) and value appraisals (perceived value of activities or outcomes) (Pekrun and Stephens, 2010). Therefore, it is suggested that control-value appraisals play a mediating role between learning environments and emotions experienced (Pekrun, 2006).

In this way, achievement emotions, as a cause to explain the behavior of students (Simonton and Garn, 2020), can be classified based on three main criteria: their valence (positive or negative), their level of activation (activating or deactivating) and the focus of the objective, that is whether it is an activity (e.g., participating in a task) or a result (e.g., winning or losing a competition) (Fierro-Suero et al., 2020a). So, emotions are specific reaction to specific tasks or situations, and not general feelings. For example, enjoyment is a positive and activating emotion focused on the activity performed. However, boredom could be considered an opposite emotion, since it is an emotion with negative valence and is deactivating also focused on the activity (for more information, see Fierro-Suero et al., 2023). Studies based on CVTAE principles have shown the importance of the emotions experienced during PE classes in explaining outcomes such as academic achievement, commitment, disruptive behaviors, PA levels or future intention to practice (Simonton and Garn, 2020; Zimmermann et al., 2021; Fierro-Suero et al., 2023).

### 1.3. Relation between SDT and emotions

Motivation and emotions separately have been shown to be consistent and effective in explaining behaviors such as PA level, future intention to practice, academic achievement or disruptive behavior in PE classes (Ulstad et al., 2016; Franco and Coterón, 2017; Simonton and Garn, 2020; Zimmermann et al., 2021; Fierro-Suero et al., 2023). However, few investigations so far have addressed the study of both constructs simultaneously in PE (Fierro-Suero et al., 2022, 2023). Given the similarity between the postulates of CVTAE and SDT, this study proposes to incorporate the emotional and motivational roles into understanding the consequences of PE classes.

It is known that emotions are omnipresent in classrooms and essential to understanding educational interactions (Meyer and Turner, 2006). In the educational environment, students will experience a type of emotion that depends on whether or not their expectations are met (Meyer and Turner, 2006). The fulfillment of expectations is mediated by conscious or unconscious evaluations of what happens to us (Pekrun and Stephens, 2010). These regulatory processes are associated with the level of satisfaction of the BPN (Ryan and Deci, 2001). That is, emotions can be understood as the result of the satisfaction or thwarting of BPN (Ryan and Deci, 2001; Flunger et al., 2013; Løvoll et al., 2020). For example, a student will experience enjoyment during the learning process if they feel competent to meet the demands of the task and value what he or she is learning (Pekrun and Stephens, 2010), as previously shown in PE classes (Leisterer et al., 2019; Fierro-Suero et al., 2020a). Thus, depending on whether teachers establish supportive or controlling interpersonal styles for the BPN in the learning environment, the BPN will be satisfied or thwarted and simultaneously autonomous motivation and positive or negative emotions will be aroused (e.g., Yoo, 2015; Bordbar, 2019). Finally, both emotions and the different types of motivational regulations have been shown to have the ability to predict the outcomes mentioned above (e.g., Fierro-Suero et al., 2022).

Recently, in different educational contexts, models based on the support of BPN have been proposed (e.g., Yoo, 2015; Bordbar, 2019; Liu et al., 2021; Zimmermann et al., 2021). These studies have advanced our understanding of the interrelationship and connection between SDT and emotional aspects. From these studies it is concluded that interpersonal style affects students emotionally and that, in turn, this has different consequences. However, most of these studies (e.g., Yoo, 2015; Bordbar, 2019; Zimmermann et al., 2021) have only considered different forms of autonomy support, ignoring the rest of BPN. On the other hand, the mediating role that BPNs can play between the teacher’s interpersonal style and emotions has not been studied so far. In conclusion, studies conducted do not take into account the full bright pathway (motivating style) of the SDT sequential model (BPN support ➔ BPN satisfaction ➔ motivation ➔ outcomes; Vasconcellos et al., 2020) or have established a chronological ordering of the motivation and emotion (e.g., Yoo, 2015).

### 1.4. The importance of gender in PE

In recent years, the importance of gender in understanding the experience of students during PE classes has been highlighted. Aspects related to the teacher’s gender and its stereotypes (Preece et al., 2022), the relationship with the teacher (van Aart et al., 2017) or the degree of physical competence (Cairney et al., 2012; van Aart et al., 2017) have been shown to affect each gender differently, ultimately impacting the students’ experience during classes. For this reason, it is necessary to take into account the gender of the students in this study, as the aforementioned factors are closely related to both SDT (Deci and Ryan, 1985; Ryan and Deci, 2017) and CVTAE (Pekrun, 2006).

### 1.5. Outcomes in PE

Two of the most studied outcomes that have a direct relationship with what is experienced in the PE classroom are academic achievement and the intention to be physically active. On the one hand, academic achievement is influenced by several factors highlighting the role of emotions (Pekrun et al., 2017; Fierro-Suero et al., 2023) and motivation (Cheon et al., 2012; Ulstad et al., 2016). Academic achievement can be defined as a “product achieved by students in educational institutions and that is normally expressed through school grades” (Fraile-García et al., 2019, p. 58). On the other hand, one of the main aim of PE should be to prepare children for a lifetime of physical activity (Leisterer et al., 2019). The intention expressed by student to practice physical activity can be a good predictor of this behavior (Ajzen, 1991). This intention has been predicted by autonomous motivation (Vasconcellos et al., 2020) and positive emotions (Fierro-Suero et al., 2023) in previous research.

### 1.6. The present study

**Figure 3:**
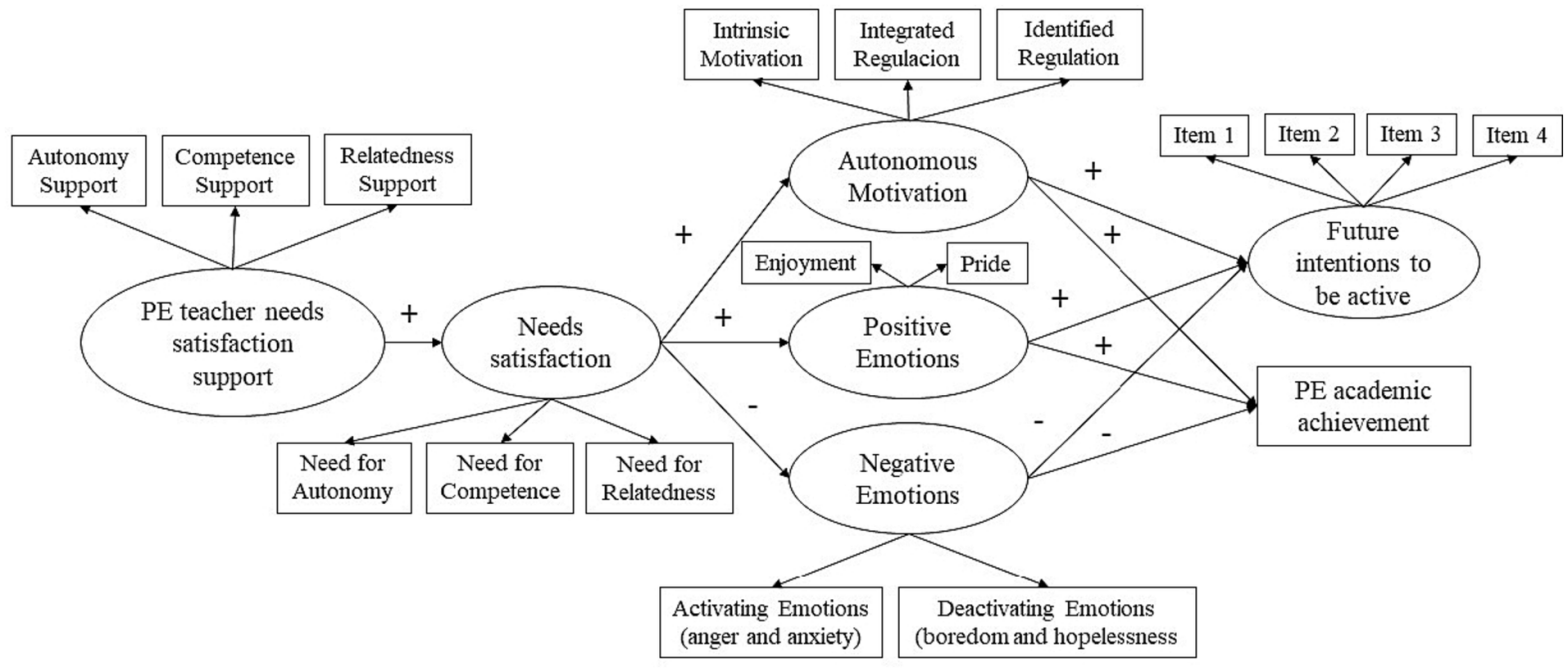
Hypothesized structural model of the associations between PE students’ perceptions of their teacher’s support for satisfaction of their needs, the satisfaction of their needs, autonomous motivation, positive and negative emotions, future intention, and PE academic achievement.

This study includes the role of positive and negative emotions in the bright pathway of SDT. This novelty represents an advance on previous studies and will help to understand to what extent the outcomes may be due to one construct or the other. Based on the established principles, the objective of this study was to examine the role that emotions and motivation play on the intention to perform future PA and on academic achievement in PE. For this, a mediated effect model was tested that suggests that the teachers’ support of the students’ BPN will satisfy the students’ BPN (hypothesis 1), and in turn, that satisfaction predicts both the autonomous motivation and the emotions (positive and negative) of the students (hypothesis 2). Finally, autonomous motivation and positive emotions will positively predict both intention to practice in the future and academic achievement, while negative emotions will negatively predict intention to practice in the future and academic achievement of students (hypothesis 3). Likewise, the mediating role of BPN satisfaction is examined, as well as motivation and emotions in these relationships (see Figure 3). In this sense, BPN satisfaction will mediate the relationship between teacher’s support for BPN and motivation and emotions, while autonomous motivation and emotions will mediate the relationship between satisfaction of BPN and intention to practice in the future and academic achievement in PE (hypothesis 4). Since SDT and CVTAE have been shown to be independent of the gender of the students (Goetz et al., 2008; Guérin et al., 2012), we hypothesized that the model including the emotions in the complete SDT sequence, is gender independent (hypothesis 5), although there could be differences in the emotions and motivational regulations experienced between the two genders.

## 2. Method

### 2.1. Design section

This research had a non-experimental quantitative, correlational and cross-sectional design (Ato et al., 2013).

### 2.2. Participants

The participants in this study were a convenience sample of 799 high school students (371 males and 428 females) aged 11–17 years (*M* = 13.16; SD = 1.17). Students were recruited from one private and four public high schools in a province in the south-west of Spain. The respondents were 253 students of first graders, 283 s graders, 207 third graders, and 56 fourth graders. PE lesson were two 60-min compulsory and coeducational per week. The PE curriculum implemented in Spain educational law is focused on the teaching of games and sports, teaching of corporal expression, development of physical and motor condition, development of health and quality of life and teaching of physical activities in the natural environment.

### 2.3. Measures

#### 2.3.1. Teacher support for basic psychological needs

The *teacher support for basic psychological needs* was assessed using the scale developed by Sánchez-Oliva et al. (2013). This scale consists of a total of 12 items, four for each BPN: *Autonomy* (e.g., “Often asks us about the activities we want to do”), *competence* (e.g., “Encourages us to trust our ability to complete the tasks well”), and *relatedness* (e.g.,” Always fosters good relationships between classmates”). Items are preceded by the stem “In my Physical Education class, the teacher…” The responses to the items are given on a Likert-type scale from 1 (Strongly disagree) to 5 (Strongly agree). Evidence for the reliability and validity of this questionnaire has been previously provided (e.g., Sevil-Serrano et al., 2020; Fierro-Suero et al., 2020b).

#### 2.3.2. Satisfaction of basic psychological needs

The *satisfaction of basic psychological needs* was assessed using the scale developed by Moreno et al. (2008). The scale consists of 12 items grouped into three sub-scales of four items each: the *need for autonomy* (e.g., “I feel very strongly that I have the opportunity to make choices about the way I exercise”), the *need for competence* (e.g., “I feel that exercise is an activity in which I do very well”), and the *need for relatedness* (e.g., “I feel very much at ease with the other exercise participants”). The scale begins with the stem “In my Physical Education class …,” and the answers are from a Likert scale ranging from 1 (Strongly disagree) to 5 (Strongly agree). Previous studies have tested this instrument’s reliability and validity (e.g., Gil-Arias et al., 2017; González-Cutre et al., 2020).

#### 2.3.3. Autonomous motivation

The Spanish version (Ferriz et al., 2015) of the *Perceived Locus of Causality Scale* (Goudas et al., 1994) was used to assess *autonomous motivation*. This scale includes 12 items encompassing three sub-scales of four items each: *intrinsic regulation* (e.g., “Because Physical Education is fun”), *integrated regulation* (e.g., “Because it is in line with my way of life”) and *identified regulation* (e.g., “Because I want to learn sports skills”). The scale begins with the stem “I take part in Physical Education …” and the items are answered on a 7-point Likert-type scale ranging from 1 (Strongly disagree) to 7 (Strongly agree). Evidence for the reliability and validity of this questionnaire has been provided in the PE context (e.g., Gil-Arias et al., 2017; Franco et al., 2021).

#### 2.3.4. Achievement emotions

*Positive and negative emotions* were evaluated with the version for *Physical Education of the Achievement Emotions Questionnaire* (Fierro-Suero et al., 2020a). This questionnaire contains 24 items grouped into six sub-scales (two positive and four negative emotions) of four items each: *Pride* (e.g., “I am proud of my participation in physical education class”), *enjoyment* (e.g., “I enjoy being in the physical education class”), *anger* (e.g., “I feel anger welling up in me during the physical education class”), *anxiety* (e.g., “I feel nervous in the physical education class”), *hopelessness* (e.g., “It is pointless to prepare for the physical education class because I am bad at it anyway”), and *boredom* (e.g., “I get bored during the physical education class”). The responses to the items are given on a Likert scale ranging from 1 (Strongly disagree) to 5 (Strongly agree). Evidence for the reliability and validity of this questionnaire has been previously provided (e.g., Fierro-Suero et al., 2022, 2023).

#### 2.3.5. Intention to be physically active

The *intention to be physically active* was evaluated with the Spanish version (Moreno et al., 2007) of the *Intention to be Physically Active Scale* (Hein et al., 2004). This scale consists of 5 items (e.g., “I would like to be physically active”) preceded by the stem “Regarding my intention to practice sport or physical activity in my free time ….”. The responses to the items are given on a Likert-type scale ranging from 1 (Strongly disagree) to 5 (Strongly agree). The reliability and validity of this questionnaire have been confirmed previously (e.g., Fernández-Espínola et al., 2020; Merino-Barrero et al., 2020).

#### 2.3.6. Academic achievement in physical education

To measure *academic achievement in PE*, the participant’s score (0–10) on their last final PE assessment was used. This score was decided by the teachers based on the evaluation criteria established in the educational laws in Spain for PE. This method has been used in previous studies (e.g., Pekrun et al., 2009; Hagen et al., 2021).

### 2.4. Procedure

This study was conducted in accordance with the ethical principles of the American Psychological Association and was approved by the Andalusian (Spain) Ethics Committee for Biomedical Research (TD-OCME-2018). First, the researchers directly contacted school administrators and school boards to inform them and request their cooperation in the research. As the students were minors, written authorization was requested from both the school and the parents of the participants. A member of the research team was present during the administration of the questionnaire. The questionnaires were answered anonymously and administered in a classroom setting. The students participated voluntarily and took approximately 30 min to complete the questionnaires. To avoid possible biases in participant’s responses, we eliminated those students who completed the test in less than half the time than the group average and those who filled in all responses with the same score.

### 2.5. Data analysis

Descriptive statistics (mean, standard deviation, skewness, kurtosis), bivariate correlations and internal consistency (Cronbach alpha and Omega coefficients) were analyzed using PROCESS MACRO version 3.0 (Hayes and Coutts, 2020) for IBM SPSS Statistics version 20 (IBM Corp. Armonk, NY, USA). We performed a hypothesis contrast test using the Fisher r to z transformation to examine whether the correlations between the hypothesized model variables were similar between males and females. In the case of finding differences by gender, we would analyze the gender invariance of the hypothesized model. The percentage of missing data was very small (< 0.05%). To examine the factorial structure of each instrument and to test the study hypotheses (see Figure 3), we used Mplus version 8 (Muthén and Muthén, 2017). We ran a mediated regression model and, to verify the fit of the models, we considered the chi-square, the Tucker-Lewis Index (TLI), the Comparative Fit Index (CFI), the Root Mean Square Error of Approximation (RMSEA), and the Standardized Root Mean Square Residual (SRMR), Average Variance Extracted (AVE) and Composite Reliability (CR). Values of TLI and CFI above 0.90 indicate an acceptable fit. For RMSEA and SRMR, values equal or below 0.08 are considered satisfactory (Hu and Bentler, 1999). For AVE and rho values above 0.50 and 0.70 indicates a good score reliability (Fornell and Larcker, 1981; Raykov, 2001). The Mardia’s coefficient was calculated (57.20) indicating multivariate non-normality in the data (Satorra and Bentler, 1994). So, the structural models were tested using maximum likelihood as the estimation method and modeling the relationships among the observed. To test the mediated or indirect effects, we used the bias-corrected bootstrap confidence interval method as implemented in Mplus. If the confidence interval does not include zero, the null hypothesis of no mediation is rejected, providing empirical support for the indirect effect.

The gender invariance testing of the model involved two hierarchically ordered steps. First, the *a priori* factor structure was fitted separately for each gender to determine the extent to which the reference model fit the data for each gender separately. Second, the configural invariance model tested the invariance of the hypothesized relationships of the model across seasons, but no invariance constraint was imposed in any parameters. This model was used as a baseline for fit comparisons against the later, more restricted model. Finally, a total invariance model addressed the equality of all parameters across gender. Thus, this model tested whether all relationships between the variables in the model remained invariant across the two genders. With the aim of assessing the fit for the models, the same modeling rationale we employed to test mediation effect of the needs and emotions was used.

Due to the number of parameters in the hypothesized model, we used means scores of three indicators of the *support for basic psychological needs (autonomy support, competence support and relatedness support)*, means scores of three indicators of the *satisfaction of the basic psychological needs (needs for autonomy, competence and relatedness)*, means scores of three indicators of *autonomous motivation (intrinsic, integrated and identified regulations)*, means scores of two indicators of *positive emotions (enjoyment and pride)*, means scores of two indicators of *negative emotions (activating negative emotions: anger and anxiety*; and *deactivating negative emotions: boredom and hopelessness)*, four items for future *intention to be physically active*, and one item for *academic achievement* (see Figure 3). So, seven observed variables were included in the model.

## 3. Results

The results of the factorial structure of the instrument used offered acceptable fit indices (see Table 1). The descriptive statistics (range, mean, standard deviations, skewness and kurtosis) and internal consistency of the study variables appear in Table 2. Descriptive statistics for all study indicators can be found in Supplementary Table S1. The participants exhibited moderate average scores, above the response scale’s nominal midpoint on all the variables (except for negative emotions). All the variables showed acceptable alpha and omega coefficients (see Table 2). All the study variables, that is, support for basic psychological needs, satisfaction of basic psychological needs, autonomous motivation, positive emotions, intention to be physically active in the future and academic achievement (except for negative emotions) were positively correlated between themselves, and all these variables were negatively correlated with negative emotions (see Table 2).

**Table 1:** Goodness-of-fit indices for the study instruments.

	*χ2*	*df*	CFI	TLI	RMSEA	SRMR	AVE	CR
Basic needs support	226.46	51	0.97	0.96	0.06	0.03	0.56	0.94
Basic needs satisfaction	283.34	51	0.94	0.92	0.07	0.06	0.52	0.93
Autonomous motivation	342.58	51	0.96	0.94	0.08	0.03	0.61	0.95
Achievement emotions	928.39	245	0.92	0.91	0.06	0.06	0.53	0.91
Future intention	13.85	2	0.96	0.96	0.08	0.02	0.50	0.80

For all χ^2^ values, *p* < 0.01; df, degrees of freedom; CFI, comparative fit index; TLI, Tucker–Lewis index; RMSEA, root mean square error of approximation; SRMR, standardized root mean residual; AVE, average variance extracted; CR, composite reliability.

**Table 2:** Descriptive statistics, internal consistency, and bivariate correlations between study variables.

Variable	1	2	3	4	5	6	7
1. Basic needs support	-						
2. Basic needs satisfaction	0.58**	-					
3. Autonomous motivation	0.57**	0.71**	-				
4. Positive emotions	0.55**	0.71**	0.73**	-			
5. Negative emotions	−0.43**	−0.58**	−0.52**	−0.64**	-		
6. Future intention	0.25**	0.50**	0.59**	0.47**	−0.34**	-	
7. Academic achievement	0.30**	0.40**	0.40**	0.40**	−0.38**	0.29**	-
Range	1–5	1–5	1–7	1–5	1–5	1–5	0–10
Mean	3.71	3.69	5.39	4.03	1.66	4.13	7.53
Standard deviation	0.85	0.71	1.27	0.82	0.59	0.90	1.56
Skewness	−0.87	−0.65	−0.90	−1.01	1.65	−1.10	−0.59
Kurtosis	0.35	0.17	0.42	0.68	3.51	0.75	−0.17
Alpha	0.92	0.87	0.94	0.89	0.87	0.77	-
Omega	0.92	0.87	0.94	0.88	0.87	0.77	-

**p < 0.001.

The results for gender differences in the correlations showed that there were no significant differences (*z* < 1.96) in the majority of the studied variables (see Table 3). However, the differences found are precisely in the relationships with the outcome variables, so we tested the hypothesized model analyses on boys and girls individually (see Figure 3).

**Table 3:** Results of values of correlation differences across gender for the study variables.

Correlation variables	Males correlation	Females correlation	z
Basic needs support – basic needs satisfaction	0.54**	0.61**	−1.47
Basic needs satisfaction – autonomous motivation	0.66**	0.72**	−1.61
Basic needs satisfaction – positive emotions	0.69**	0.70**	−0.27
Basic needs satisfaction – negative emotions	−0.53**	−0.60**	−1.45
Autonomous motivation – future intention	0.54**	0.60**	−1.25
Positive emotions – future intention	0.32**	0.53**	−3.63**
Negative emotions – future intention	−0.25**	−0.36**	1.70
Autonomous motivation – academic achievement	0.33**	0.46**	−2.17*
Positive emotions – academic achievement	0.33**	0.45**	−1.99*
Negative emotions – academic achievement	−0.34**	−0.41**	−1.14

*p < 0.05; **p < 0.001.

**Figure 4:**
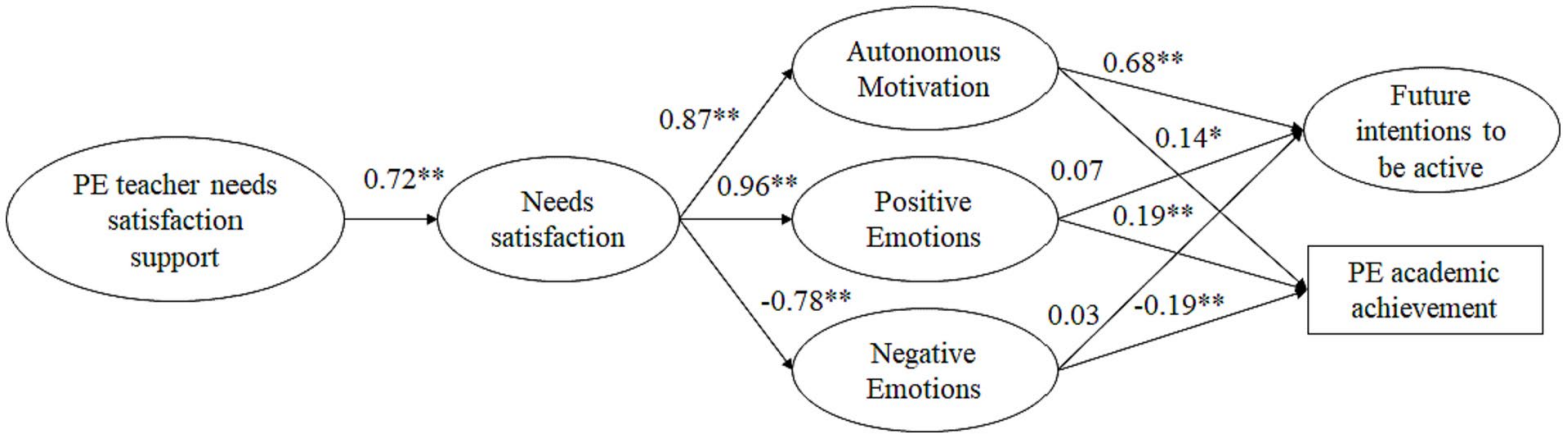
Gender-invariant structural. All coefficients are standardized. Factor indicators are not shown for reasons of simplicity of presentation. **p < 0.01; *p < 0.05.

The hypothesized structural model for boys [χ^2^ (125) = 474.91, *p* = 0.001, TLI = 0.900; CFI = 0.911; SRMR = 0.055; RMSEA = 0.087] and girls [χ^2^ (125) = 524.89, *p* = 0.001, TLI = 0.901; CFI = 0.919; SRMR = 0.050; RMSEA = 0.086] showed an adequate fit to the data. Next, the configural invariance model was tested by analyzing the invariance of the factor structure without putting restrictions on the parameters, and the fit was satisfactory [χ^2^ (250) = 906.77, *p* = 0.001, TLI = 0.900; CFI = 0.915; SRMR = 0.056; RMSEA = 0.061]. Consequently, this model was used as a baseline for comparison with the total invariance model, where the restriction of equality in all parameters is assumed in the two samples. The total invariance model had an adequate fit [χ^2^ (277) = 943.13, *p* = 0.001, TLI = 0.903; CFI = 0.913; SRMR = 0.078; RMSEA = 0.059]. Differences not larger than 0.01 between TLI and CFI values are considered an indication of negligible practical differences (Cheung and Rensvold, 2002). For RMSEA and SRMR, values equal to or lower than 0.08 are optimal (Cole and Maxwell, 1985). The results showed that the models compared showed acceptable fit indices, with no significant differences between the model without restrictions and the model with total restriction, which supports the existence of model invariance in both groups. The standardized parameter estimates are shown in Figure 4.

The results revealed partial support for the hypothesized model. Perceptions of *PE teacher support for satisfaction of psychological needs* were positively and highly related to *satisfaction of basic psychological needs*. In line with the proposed model, there was a significant and positive path between *satisfaction of needs* and *autonomous motivation*, and *positive emotions*, and negatively with *negative emotions*. *Autonomous motivation* was positively and moderately related to *intentions to be physically active in the future* and weakly related to *PE academic achievement*. The paths between *positive and negative emotions* with *future intention* were not significant. Finally, *positive emotions* were weakly significant and positively related to *PE academic achievement*, whereas the relation between *negative emotions* and *PE academic achievement* was significant and negative (see Figure 4). The results of the proposed model significantly predicted 51% of the variance in reported *intention to be physically active in the future* and 22% of the variance in reported *PE academic achievement*.

Finally, we analyzed the indirect effects (IE) of the *PE teacher support for satisfaction of needs* on *intentions to be active in the future* and *PE academic achievement* through *satisfaction of needs, motivation and emotions (positive and negative)*. The IE on *intentions to be active in the future* through *satisfaction of needs* and *autonomous motivation* was positive and statistically significant (IE = 0.26; bootstrap CI 95% = [0.21, 0.31]). The IE on *PE academic achievement* through *satisfaction of needs* and *autonomous motivation* was also positive and statistically significant (IE = 0.16; bootstrap CI 95% = [0.08, 0.24]). The *IE on PE academic achievement* through *satisfaction of needs* and *positive emotions* was positive and statistically significant (IE = 0.10; bootstrap CI 95% = [0.01, 0.18]). Finally, the IE on *PE academic achievement* through *satisfaction of needs* and *negative emotions* was also positive and statistically significant (IE = 0.12; bootstrap CI 95% = [0.06, 0.18]). These results provide total support for the indirect effect of the *PE teacher’s support for satisfaction of needs* on *intentions to be physically active in the future* and *PE academic achievement* through s*atisfaction of needs* and *autonomous motivation*, and the partial support through *satisfaction of needs* and *emotions* on *PE academic achievement* but not on *intentions to be physically active in the future*.

## 4. Discussion

The objective of this study was to find the role that emotions and motivation in PE classes play on academic achievement and the intention to practice PA in the future. To date, studies conducted to explain these outcomes have focused mainly on student motivation (e.g., Ulstad et al., 2016; Franco and Coterón, 2017; Castillo et al., 2020). However, despite the fact that emotions give us important information about human behavior (Meyer and Turner, 2006) and the significant impact on the educational context (Ramirez-Arellano et al., 2019; Järvenoja et al., 2020), there have been few studies of this construct. In recent years, different investigations have shown that both CVTAE at an emotional level (Simonton and Garn, 2020; Zimmermann et al., 2021; Fierro-Suero et al., 2023) and SDT at the motivational level (Franco and Coterón, 2017; Castillo et al., 2020) are able to explain outcomes in PE classes. Currently, there are few investigations that jointly analyze the motivational and emotional roles without prioritizing one construct above the other in PE classes. For this, in this study, the integration of emotions in SDT has been proposed following the common postulates between CVTAE and SDT. Thus, the hierarchy of one construct over the other is avoided, allowing for both clearly influencing human behavior (Reeve, 2010).

The analysis of the structural equations model confirmed hypothesis 1 since the support of the BPN by the teacher in the learning environment acted as a predictor of the satisfaction of the students’ BPN. These results have been widely supported by previous scientific literature (Vasconcellos et al., 2020). For the study’s second hypothesis, the proposed model showed that satisfaction of BPN was a significant predictor of both autonomous motivation and emotions (positive and negative), confirming hypothesis 2. Different studies have shown that satisfaction of BPN provokes more self-determined motivations, as established by the SDT (e.g., Haerens et al., 2015; Franco and Coterón, 2017). Although this theory does not explicitly include the emotional role in its sequence, it does state that the satisfaction of BPN produces positive emotions (Ryan and Deci, 2001), which has been shown in education in general (Flunger et al., 2013) and in PE in particular (e.g., Leisterer et al., 2019; Løvoll et al., 2020; Fierro-Suero et al., 2020a, 2023). In this way, as established by the CVTAE depending on the control and value appraisals, evaluations that are closely related to the BPN, some emotions or others will be generated (Pekrun and Stephens, 2010).

Hypothesis 3 of the study has been partially fulfilled. To date, research has shown that both motivation (Ulstad et al., 2016; Franco and Coterón, 2017; Castillo et al., 2020) and emotions (Simonton and Garn, 2020; Fierro-Suero et al., 2023) independently, were able to predict academic achievement in PE and intention to be active in the future. In this sense, the results of this study have shown that the intention to be physically active in the future was only predicted by autonomous motivation, with the effect of emotions experienced during PE classes having a lesser effect. These results are consistent with those found by Løvoll et al. (2017) who showed that, despite the fact that positive emotions and intrinsic motivation both acted as predictors of the choice of future PA, by including both variables in the regression model the emotional effect is completely reduced. The intention of future PA is based on a subjective perception that requires a reflective process. On the one hand, this process is closely related to the orientation, management and persistence over time of the behavior, that is, to the definition of motivation (Reeve, 2010). On the other hand, it requires more extensive cognitive processing and, therefore, a deliberation associated more with motivational behaviors than with emotional ones (Roseman, 2013). Another aspect to consider is that emotions are situation specific, the perceptions of control-value that one develops in a specific context are what form an emotional response to that specific context (PE in this case), therefore, extracurricular PA may be quite different from PE experiences. Momentary emotions are associated with specific behaviors, such as anger with aggressive reactions or boredom with disinterest, which can affect academic achievement. The assessment of practicing physical-sports activity could be too generic a behavior to be affected by the achievement emotions experienced during the classes. Along these lines, Løvoll et al. (2017) found that emotions played a different role in predicting outdoor PA or in choosing a sport. Thus, it makes sense to think that the intention to practice in the future is explained more by autonomous motivation than by emotions, as the results obtained have indicated.

For its part, academic achievement is an objective measure provided by teachers that does not require the reflective process on the part of the students mentioned above. So far, the importance of emotions on academic achievement has been shown (Pekrun et al., 2009; Fierro-Suero et al., 2023) as they directly affect the specific behaviors of students during classes (Roseman, 2013; Simonton and Garn, 2020) impacting on the teachers’ assessment of them. Although, to a lesser extent than emotions, autonomous motivation also acted as a predictor of academic achievement, as confirmed by previous studies (Cheon et al., 2012; Ulstad et al., 2016). Therefore, it could be concluded that motivation plays a more significant role in explaining the future intention to practice PA and emotions play a more decisive role in students’ academic achievement. This may be because motivational behavior is more deliberate and prolonged (Roseman, 2013) and can be extrapolated toward its intention for the future. However, emotional behavior, being more sporadic, has a more short-term effect, which can affect behaviors that occur over a short space of time to a greater extent.

Therefore, the results of the structural equation model and the indirect effects confirm hypothesis 4. These show that the sequence proposed, in which the emotional and motivational roles are integrated, following the principles of the CVTAE and SDT, is consistent and opens an avenue for future research. It will be interesting to include into the model in the future other variables related to both theories’ postulates, such as self-efficacy expectations, attributions of achievement, the teacher’s controlling style, thwarting of BPN, etc. Advancing along these lines will mean going into greater depth for the interrelation between motivation and emotion and the repercussions of these during the classes.

The results confirmed that the proposed model is gender-independent, confirming hypothesis 5. However, it is interesting to highlight the gender differences found in the relationships (correlations differences across gender) of motivation and emotions to the two outcomes studied (intention to practice PA in the future and academic achievement). These results seem to indicate that, as has recently been shown (Fierro-Suero et al., 2023), the imprint of what is experienced during the classes conditions the behavior of girls more than boys. As the authors argue, this could be due to various factors such as the difference in character between boys and girls (Chaplin and Aldao, 2013), the role of the teacher and their stereotypes (Preece et al., 2022), or the lower level of after-school PA in girls (Lodewyk and Muir, 2017). This last factor could allow the extracurricular PA experience, more notable in boys than in girls, to compensate for the assessment made of sport and PA (Fierro-Suero et al., 2023). Thus, for those students who do not do after-school PA, PE is the most important physical experience (Shephard, 2008) and therefore, their assessment of what happened may condition the outcomes studied to a greater extent. Expanding the outcomes analyzed taking into account both constructs should be a priority. This would allow the preparation of interventions that help to find effective and efficient strategies to improve educational quality, which should be a priority for researchers and teachers.

### 4.1. Practical implications

The present findings have important implications for current theorizing, research, and practice on motivational and emotional teaching. Emotions have been integrated into SDT model, which represents a significant advance compared to previous research. Simultaneous examination of motivations and emotions in students has shown how it is possible to differentiate in terms of individual perceptions (Ramirez-Arellano et al., 2019; Van Roekel et al., 2019) as well as the consequences of these, despite the difficulty of differentiating between motivational and emotional aspects of teaching styles (Meyer and Turner, 2006). Therefore, having a supportive interpersonal motivational style helps to improve both students’ motivations and emotions. To improve interpersonal style, it is recommended to consult the recent classification made by a wide panel of international experts (Ahmadi et al., 2023). Based on the present results and previous studies (e.g., Fierro-Suero et al., 2020a; Zimmermann et al., 2021) it would be interesting to complement the motivational style (based on SDT) with the recommendations on how to improve emotional perception in PE classes from the latest studies developed (e.g., Simonton and Garn, 2018, 2020; Fierro-Suero et al., 2023).

### 4.2. Limitations and future work

This study has some limitations. First, we used a correlational and cross-sectional design, which prevents us from considering causal relationships. Second, most of the information collected was self-reported, with the exception of academic achievement, and no information was collected from the teacher on his or her perception of support for the students’ basic psychological needs. Due to the number of variables collected, in the hypothesized model we have had to group the emotions according to the degree of activation and valence. Although this may represent a loss of value, at the same time it provides a basis for future research to address these relationships more specifically. Finally, we have only considered the bright pathway of the SDT. It could be interesting in the future to carry out studies from the SDT dark pathway (demotivating style) or to consider other variables related to CVTAE, the gender of teachers, the age range between teachers and students, as well as longitudinal studies, which would allow us to contrast it with the results obtained in this study.

## 5. Conclusion

In conclusion, this study has shown the importance of including both motivation and emotions to understand the consequences of what happens in PE classes. Thus, when jointly studying motivation and emotion, following the theoretical principles of the SDT, it has been found that motivation plays a more significant role in explaining the intention to practice PA in the future outside school. However, although motivation is also important, the emotions experienced by students explain their academic achievement to a greater extent. It is essential to continue advancing along this line as a first step to establishing more effective strategies to improve educational quality.

## Data availability statement

The original contributions presented in the study are included in the article/Supplementary material, further inquiries can be directed to the corresponding author based on reasonable request due to ethical restrictions.

## Ethics statement

The studies involving humans were approved by Andalusian (Spain) Ethics Committee for Biomedical Research (TD-OCME-2018). The studies were conducted in accordance with the local legislation and institutional requirements. Written informed consent for participation in this study was provided by the participants’ legal guardians/next of kin. Written informed consent was obtained from the minor(s)’ legal guardian/next of kin for the publication of any potentially identifiable images or data included in this article.

## Author contributions

IC, BA, and PS-L: conceptualization, supervision, and validation. SF-S: data curation. SF-S and IC: formal analysis, funding acquisition and writing – original draft. SF-S, IC, and BA: investigation and software. SF-S and PS-L: methodology. SF-S, IC, and PS-L: project administration. SF-S, IC, BA, and PS-L: resources, visualization, and writing – review and editing. All authors have read and agreed to the published version of the manuscript.
