# Supplementary material for: The role of motivation and emotions in physical education: understanding academic achievement and the intention to be physically active
Source: Front Psychol. 2023 Sep 20;14:1253043. doi: 10.3389/fpsyg.2023.1253043 (PMC10548266; doi:10.3389/fpsyg.2023.1253043)
Supplement: Supplementary file 1 [file Table_1.docx]

| **Variable** | **1** | **2** | **3** | **4** | **5** | **6** | **7** | **8** | **9** | **10** | **11** | **12** | **13** | **14** | **15** |
| --- | --- | --- | --- | --- | --- | --- | --- | --- | --- | --- | --- | --- | --- | --- | --- |
| 1. Autonomy support | - |  |  |  |  |  |  |  |  |  |  |  |  |  |  |
| 2. Competence support | .71** | - |  |  |  |  |  |  |  |  |  |  |  |  |  |
| 3. Relatedness support | .65** | .74** | - |  |  |  |  |  |  |  |  |  |  |  |  |
| 4. Autonomy satisfaction | .60** | .57** | .48** | - |  |  |  |  |  |  |  |  |  |  |  |
| 5. Competence satisfaction | .32** | .43** | .39** | .56** | - |  |  |  |  |  |  |  |  |  |  |
| 6. Relatedness satisfaction | .26** | .32** | .43** | .38** | .54** | - |  |  |  |  |  |  |  |  |  |
| 7. Intrinsic motivation | .46** | .61** | .51** | .59** | .59** | .45** | - |  |  |  |  |  |  |  |  |
| 8. Integrated regulation | .38** | .47** | .43** | .55** | .65** | .46** | .78** | - |  |  |  |  |  |  |  |
| 9. Identified regulation | .39** | .56** | .50** | .52** | .56** | .42** | .80** | .81** | - |  |  |  |  |  |  |
| 10. Pride | .34** | .45** | .38** | .50** | .62** | .42** | .59** | .57** | .56** | - |  |  |  |  |  |
| 11. Enjoyment | .47** | .57** | .49** | .58** | .61** | .45** | .72** | .66** | .63** | .70** | - |  |  |  |  |
| 12.Negative activating emotions | -.16** | -.26** | -.25** | -.27** | -.42** | -.40** | -.34** | -.32** | -.26** | -.37** | -.39** | - |  |  |  |
| 13. Negative deactivating emotions | -.40** | -.51** | -.45** | -.48** | -.55** | -.42** | -.61** | -.52** | -.54** | -.57** | -.73** | .57** | - |  |  |
| 14. Future intention | .19** | .23** | .26** | .35** | .54** | .34** | .49** | .63** | .54** | .41** | .45** | -.24** | -.37** | - |  |
| 15. Academic achievement | .22** | .31** | .28** | .29** | .40** | .30** | .38** | .39** | .34** | .34** | .40** | -.26** | -.41** | .29** | - |
| Range | 1–5 | 1–5 | 1–5 | 1–5 | 1–5 | 1–5 | 1–7 | 1–7 | 1–7 | 1–5 | 1–5 | 1–5 | 1–5 | 1–5 | 0–10 |
| Mean | 3.09 | 3.98 | 4.06 | 3.05 | 3.80 | 4.21 | 5.42 | 5.11 | 5.63 | 4.01 | 4.05 | 1.70 | 1.63 | 4.13 | 7.53 |
| Standard Deviation | 1.02 | .91 | .92 | .90 | .85 | .86 | 1.33 | 1.50 | 1.27 | .87 | .90 | .65 | .68 | .90 | 1.56 |
| Skewness | -.36 | -1.07 | -1.16 | -.23 | -.77 | -1.38 | -.95 | -.66 | -1.09 | .84 | .63 | 1.51 | 1.75 | -1.10 | -.59 |
| Kurtosis | -.64 | .75 | 1.06 | -.28 | .52 | 1.67 | .48 | -.32 | .95 | .-.95 | -1.09 | 2.91 | 3.86 | .75 | -.17 |

**Supplementary Table 1**. Descriptive statistics, internal consistency and bivariate correlations between study sub-scales.

Note: ** *p* < .001
